# Skin lamellar bodies: a unique set of lysosome-related organelles

**DOI:** 10.3389/fcell.2025.1597696

**Published:** 2025-06-09

**Authors:** Sarmistha Mahanty

**Affiliations:** ^1^ Amity Institute of Biotechnology, Amity University Haryana, Gurugram, India; ^2^ Amity Institute of Integrative Sciences and Health, Amity University Haryana, Gurugram, India

**Keywords:** keratinocytes, differentiation, lamellar body, secretory lysosomes, lysosome-related organelles, skin barrier

## Abstract

Skin lamellar bodies (LBs) are crucial for forming and renewing the protective skin barrier, which regulates the body’s internal environment and integrity. LB dysfunction is associated with severe disease conditions such as atopic dermatitis, Netherton syndrome and Harlequin ichthyosis, among others. Despite its importance in human physiology, the intracellular origin and biogenesis mechanism of LBs remain largely unknown. LBs are lysosome-related organelles (LRO), a group of cell type-specific organelles having unique structures, cargo content, and function. Classical LROs such as melanosomes, lung lamellar bodies and Weibel-Palade bodies share overlapped molecular machinery/mechanisms and are co-affected in genetic disorders like Hermansky-Pudlak syndrome (HPS) or Chédiak-Higashi syndrome (CHS). In contrast, LBs contain a diverse array of protein and lipid cargo that are notably different from those found in other LROs, and LBs are not reported to be affected in HPS/CHS. LBs form in an advanced differentiation state of keratinocytes while cells are experiencing high ions and low nutrients in their exterior, the plasma membrane (PM) undergoing modifications, and intracellular organelles starting to disappear. This article discusses atypical conditions of LB biogenesis in comparison to classical LROs, which may potentially guide future research on LB biogenesis.

## Highlights

• Skin lamellar bodies (LBs) regulate the formation of the skin barrier.
• LBs are classified as Lysosome Related Organelles (LROs)
• LBs are not affected by congenital disorders that impact other LROs.
• LBs are produced in an altered physiological state of keratinocytes.
• Keratinocyte lysosomes likely contribute to LB biogenesis/maturation.

## 1 Introduction

Intracellular organelles adapt to support specific cellular functions, which contributes to tissue specificity. LROs are examples of endo-lysosomal adaptations contributing to specialized functions of resident tissues. They share lysosomal cargo and membrane proteins, maintain an acidic internal environment, transport cell-type-specific cargoes, and exhibit distinct shapes and functions (Dell'Angelica et al., 2000; Delevoye et al., 2019). Typical examples of LRO include melanosomes in melanocytes, providing protection from ultraviolet light; lung lamellar bodies of alveolar Type II cells, which stabilize lung alveoli and reduce surface tension; and Weibel-Palade bodies (WPBs) in endothelial cells, which play a crucial role in blood clotting, among many others (Bowman et al., 2019).

**FIGURE 1:**
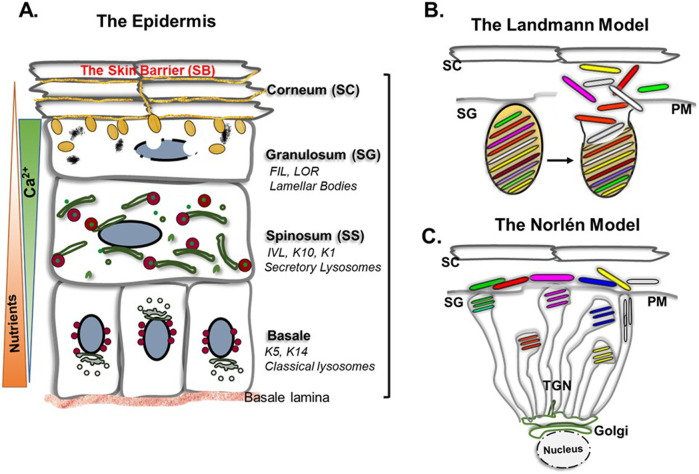
**(A)** Schematic of keratinocyte differentiation stages represented by epidermis sublayers called as stratum basale, spinosum, granulosum and corneum. Proliferative stem cells in the stratum basale gradually differentiate and move upwards. Increasing calcium and decreasing nutrients in the upper layers influence differentiation. Differentiation stages are distinguished by unique morphology and marker protein expression: the basal layer keratinocytes express Keratin5/14 (K5/K14), early differentiating spinosum layer express involucrin (IVL), keratin1/10 (K1/K10) and, advanced differentiation stage in the granulosum layer express filaggrin (FIL), and loricrin (LOR). Intracellular organelles, specifically the Golgi apparatus and lysosomes modify during differentiation. The Golgi apparatus disperses as small ministacks and establishes physical apposition/contact with lysosomes that facilitates the transport of Golgi cargoes, generating secretory lysosomes in the stratum spinosum layer (Mahanty et al., 2024). The stratum granulosum layer contains skin LBs (yellow) that secrete cargoes between the granulosum and corneum layer (SG-SC junction). Differentiation ends with the formation of corneocytes, that are metabolically arrested keratinocytes, devoid of intracellular organelles. Corneocytes along with the LB secreted components form the skin barrier on the top. The barrier is renewed through desquamation which is regulated by LB secreted enzymes. **(B)** Illustration of LB structure/morphology and secretion mechanism as described in Landmann model (Landmann, 1986). Defining LBs as discrete structures that carry wide array of cargoes into discrete disks like structures. Different colours inside these disks representing separate cargoes, packed in single LB. These disks are secreted in the SG-SC junction, upon fusion of LBs with the PM **(C)**. Representation of LB structure according to the Norlén model. It describes LBs as continuous tubulovesicular structures that span from the TGN to the PM and secrete separate cargoes in the SG-SC junction (Norlén, 2001). The bulb regions of the TGN with separate cargoes representing the presence of separate pool of LBs is based on the study by (Ishida-Yamamoto et al., 2004), and is not explained in the Norlén model. During secretion, a single fusion event forms a continuous sheet in the SG-SC junction. Please note that model B, and C is representing events in a granulosum layer cells, as LBs are enriched in this layer. TGN = Trans Golgi network; SC = stratum corneum; SG = stratum granulosum, PM = plasma membrane.

Skin LBs (also known as lamellar granules, Odland bodies, and epidermal LBs) are the LROs of keratinocytes providing functional components of the skin barrier (SB) (Odland, 1960; Elias and Feingold, 2005; Feingold, 2012). SB is the hydrophobic, selectively permeable, and immunologically active outermost skin layer that combats environmental challenges and protects internal organs (Madison, 2003; Elias, 2007; Proksch et al., 2008). Keratinocytes are the main cells of the skin epidermis that undergo a step-wise differentiation process in response to the epidermal calcium gradient and generate epidermis sublayers, arranged as proliferative basal layer ‘stratum basale’ in the base of the dermis, to gradually differentiating upper layers called stratum spinosum (SS), granulosum (SG), and corneum (SC) (illustrated in Figure 1A) (Bikle et al., 2012; Mahanty and Setty, 2021). Each sublayer represents a unique differentiation state that ends with terminal differentiation in the SC, and keratinocytes are now called corneocytes devoid of intracellular organelles, having cornified PM envelope and act as the structural components of the SB (Évora et al., 2021). Corneocytes remain embedded in a mixture of functional components secreted by LBs present in the granulosum layer, and together establish the functional barrier in the SC (Figure 1A) (Elias et al., 1998; Marks, 2004; Proksch et al., 2008; Menon et al., 2018). LB deformities result in dysfunctional SB which is associated with several socio-economically significant diseased conditions, including atopic dermatitis, Netherton syndrome and Harlequin ichthyosis (Fartasch et al., 1999; Scott et al., 2013; Elias and Wakefield, 2014; Le Lamer et al., 2015).

Despite their importance in human health, the biogenesis mechanism of LBs is largely unknown due to a lack of fundamental/molecular studies. Most of the identified LROs share significant similarities in cargo content and are generated through overlapped biogenetic machinery/molecular mechanisms (Marks et al., 2013; Banushi and Simpson, 2022). Exceptionally, skin LBs are significantly different in cargo content, cellular status they are produced, and also likely the molecular machinery involved (Elias et al., 1998; Ridsdale et al., 2011; Mahanty and Setty, 2021). Thus, modeling classical LRO/s might not help understanding LB biogenesis. In the previous review article, we discussed the possible biogenesis mechanism of skin LBs, represented in three different models (Mahanty and Setty, 2021). Our recent research finding (Mahanty et al., 2024) additionally support our ideas on LB maturation from a lysosomal precursor, which is elaborated in this article. Moreover, the unique characteristics of LBs in comparison to other LROs are discussed.

## 2 LBs regulate skin barrier development/homeostasis

**FIGURE 2:**
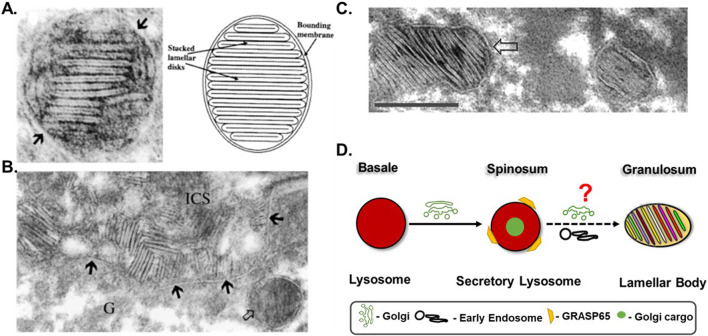
LB morphology and hypothetical maturation model: **(A,B)** The images were taken from (Madison, 2003). **(A)** The morphology of a single LB from the mouse epidermis shows characteristic disk structures. Arrows pointing to the limiting membrane of the LB. Original magnification ×,300,000. A lower-magnification image of this LG was published in (Madison et al., 1987). Copyright permission were taken from both Madison (2003) and Madison et al. (1987). **(B)** Appearance of LB-secreted disks in the intercellular space (ICS) between granulosum and corneum layer cells. Arrows pointing to the plasma membrane of a granular cell (G). Original magnification ×,125,000. **(C)** Electron microgram of a LB from human *ex vivo* skin epidermis showing similar disk structures. The image is taken with permission from Menon et al. (2018). The arrow is pointing to the limiting membrane of the LB. Scale bar = 200 nm. **(D)** Possible biogenesis/maturation mechanism of LBs through lysosomal maturation. During differentiation, the conventional lysosomes in the basal layer modify into secretory lysosomes through the Golgi input in the spinosum layer (Mahanty et al., 2024). These lysosomes likely further mature into LBs in the granulosum layer upon receiving additional input from the Golgi and endocytic compartments. The ‘?’ indicates unknown molecular machinery involved in LB maturation.

The terminally differentiated keratinocytes, or corneocytes, are the structural components that remain embedded in functional components secreted by LBs and together form the functional SB (Madison et al., 1987; Madison, 2003; Menon et al., 2018). LBs predominantly present in the granulosum layer cells and secrete barrier lipid precursors, such as glucosylceramide, phospholipids, sphingomyelin, cholesterol; lipid hydrolases such as β-glucocerebrosidase, sphingomyelinase; AMPs such as cathelicidin, LL-37; and proteases such as cathepsins and kallikreins KLK5/7/14 that help in SB renewal (Elias and Feingold, 2005; Feingold, 2012). These cargoes are secreted to the SG-SC junction, where the lipid precursors are digested by the co-secreted enzymes to form barrier lipids (Figures 2A,B) (Elias, 1981; Freinkel and Traczyk, 1985; Wertz, 1992; Madison, 2003; Elias and Feingold, 2005). The metabolites of lipid digestion also contribute additively to barrier formation, for instance, phospholipid digestion produces fatty acids and glycerol, which respectively contribute to maintaining an acidic pH of 5.5 in the SC and skin hydration; and intermediates of cholesterol digestion help in desquamation (Elias et al., 1984; Fluhr et al., 2001; Feingold et al., 2007). SB is renewed on a monthly basis by the desquamatory proteases secreted from LBs and replaced by a new batch of cells entering differentiation from the basale layer (Bikle et al., 2012; Hänel et al., 2013).

Mechanical damage to the skin destabilizes the SB, resulting in the loss of the epidermal calcium gradient. These changes are sensed by SG cells that trigger the regulated secretion of the existing pool of LBs (Menon et al., 1994; Elias et al., 1998; Denda et al., 2003). At the same time, it also promotes keratinocyte differentiation and the formation of new LBs (Menon et al., 1992; Proksch et al., 1993; Menon et al., 1994; Feingold et al., 2007). Thus, SB formation, keratinocyte differentiation and LB biogenesis regulate each other in a positive feedback loop, which is disrupted in skin disorders and wounding. Hence, LBs are crucial for SB development/homeostasis.

## 3 Debated LB shape/morphology

To restore a damaged SB, the simultaneous release of all the barrier components (LB cargoes) is necessary, which would be influenced by the LB’s shape/size and morphology (Grubauer et al., 1989; Menon et al., 1992). There is debate on skin LB morphology and, thus, on their secretion mechanism. Two different models exist on this: the Landmann model, which describes LBs as discrete organelles that carry a multitude of cargoes into distinct disks (Figures 1B, 2A,C) (Landmann, 1986; Madison, 2003). Upon LB fusion with the PM during exocytosis, these disks, containing separate cargoes, are released into the SG-SC junction (Figures 1B, 2B). The released disks then fuse together to form a continuous structure. This model would require several checkpoints and sophisticated machinery to control multiple fission/fusion steps involved. In the contrary, the Norlén model also known as the “membrane folding model”, describes LBs as part of a continuous network that extends from the TGN to the PM, and exocytose as a uniform sheet (Figure 1C). This model is biologically favorable as it maintains membrane continuity and involves fewer fission and fusion steps (Norlén, 2001; den Hollander et al., 2016). Although the Norlén model does not explain the mechanism of cargo packaging, an immuno-electron microscopy study provides insights showing five different LB cargoes appeared as separate aggregates in the bulb regions of the TGN, suggesting the possibility of the presence of different LB pools containing distinct cargoes (Ishida-Yamamoto et al., 2004). Thus, in the Norlén model LB biogenesis may depend on specific changes at the TGN, while LBs maturation in Landmann model requires a precursor organelle.

Although the LB morphology significantly differs between these models, the presence of characteristic cargo-containing disks is common in both and these disks are present in both human and mouse LBs (Figures 2A–C). There are multiple questions associated with characteristic disk structures: a) how are cargoes packed into these disks? b) what are these disks made up of? c) whether these disks are free or attached to the limiting membrane of LBs? d) in what proportion separate cargo-containing disks are loaded into individual LBs? (if LBs are discrete) e) in what order these disks are arranged within the LBs (if LBs are discrete)? f) how the formation of these disks and LB appearance is related? Nonetheless, while the secretion machinery of LROs typically involves Rab27a, CD63, VAMPs, syntaxins, and small GTPases, nothing has yet been identified for LBs (Raposo et al., 2007; Marks et al., 2013; Delevoye et al., 2019). Altogether, resolving LB morphology would significantly lead us forward in understanding its biogenesis mechanism.

## 4 Insights into the possible mechanism of LB biogenesis

LROs are formed through a unique amalgamation of the endo-lysosomal and secretory pathways. They share features similar to lysosomes, and most, if not all, undergo regulated secretion similar to secretory granules (SGs) (Delevoye et al., 2019). While some, such as melanosomes and lung lamellar bodies, originate respectively from the early and late endosomes and acquire secretory input from the Golgi apparatus during maturation, others, such as WPBs, originate from the trans-Golgi network (TGN) and mature upon receiving endosomal input (Luzio et al., 2014; Bowman et al., 2019; Delevoye et al., 2019). Despite the fact that LBs are not co-affected with other LROs in congenital disorders CHS or HPS, their biogenesis still likely involves endo-lysosomal and secretory pathways (Boissy and Nordlund, 1997; Dell'Angelica et al., 2000; Huizing et al., 2008; Mahanty and Setty, 2021). The reported tubulovesicular morphology of LBs resembling the cross-section of the TGN, and inhibition of LBs with Golgi apparatus inhibition suggests their TGN origin (Madison et al., 1998; Tarutani et al., 2012; Yamanishi et al., 2019). Therefore, the biogenesis mechanisms of TGN-derived WPBs and SGs may offer valuable insights into the formation of LBs.

The biogenesis of WPBs and SGs starts with major cargo accumulation at the TGN. von Willebrand factor (vWF), the primary cargo of WPBs accumulates by multimerization which determines the size/shape of these organelles critical for their functioning (Michaux and Cutler, 2004; Michaux et al., 2006). Similarly, in neuroendocrine cells, the biogenesis of SGs depends on the accumulation of peptide hormones (Tooze and Stinchcombe, 1992; Tooze, 1998). Multiple mechanisms, for instance, the role of specific receptors in the accumulation or the interaction of already accumulated cargo to the TGN membrane were hypothesized (Tooze, 1998). Receptor-independent mechanisms such as slightly acidic pH at the TGN, high calcium concentrations at the TGN, liquid-liquid phase separation, and protein-protein interactions may also play primary or additive roles (Tooze and Stinchcombe, 1992; Parchure et al., 2022; Campelo et al., 2023). Nonetheless, cargo accumulation ‘only’ may not be sufficient, and the role of other adaptor proteins such as AP1 and clathrin, are essential requisites for the biogenesis of both WPBs and SGs (Lui-Roberts et al., 2005; Burgess et al., 2011).

The hypothesis that a similar biogenesis mechanism could generate skin LBs through “primary cargo accumulation” has key limitations. First, this mechanism is described in normal proliferative cells, while skin LBs are produced in a physiologically modified state of keratinocytes (described below) where this mechanism may not be relevant. Second, the primary cargo vWF is exclusive to WPBs, whereas glucosylceramide, the primary cargo of LBs, is shared with lysosomes. Third, vWF is secreted in its functional form, but glucosylceramide, as a precursor, must be processed by the enzyme β-glucocerebrosidase to become ceramide, the barrier lipid (Holleran et al., 1993; Holleran et al., 1994). This indicates that glucosylceramide secretion must at least be coordinated with β-glucocerebrosidase secretion, which is also a lysosomal enzyme. Overall, these facts support our idea that skin LBs likely mature from a lysosomal precursor.

## 5 Insights into the biogenetic machinery of LBs

Although the fundamental trafficking mechanisms regulating LB biogenesis are largely unknown, experimental evidence is ample supporting active contribution of the Golgi, early endocytic organelles, and late endosome/lysosomes, as reviewed before (Mahanty and Setty, 2021). Glucosylceramide, the major cargo of LBs is synthesized in the Golgi by the enzyme glucosylceramide synthase (GCS). GCS expression works in a linear fashion with LB biogenesis (Madison et al., 1998). Unsurprisingly, functional inhibition of the Golgi apparatus using brefeldin-A or by the inhibition of Golgi pH regulator GPR89 blocks LB biogenesis (Madison and Howard, 1996; Tarutani et al., 2012). Similarly, lysosomal dysfunction due to mutation in β-Glucocerebrosidase in Gaucher’s disease and, acid sphingomyelinase dysfunction in Niemann pick disease are associated with LB deficient ichthyosis conditions and barrier dysfunction, suggesting the importance of lysosomes in LB biogenesis (Holleran et al., 1994; Schmuth et al., 2000). The critical role of shared molecular pathways involving molecules such as the biogenesis of lysosome-related organelles complex1, 2, 3 (BLOC1, BLOC2, BLOC3), small GTPases like RAB32/38, Rab9, SNAREs such as STX13, VAMP7 are defined for most of these LROs, however, disease models or available knockout mouse models for these molecules do not provide much insight on skin LB dynamics (Raposo et al., 2007; Jani et al., 2016; Bowman et al., 2019; Delevoye et al., 2019; Banushi and Simpson, 2022). Moreover, the genetic disorder HPS caused by mutations in HPS genes and/or mutation *in BLOC*/s, and CHS caused by a mutation in LYST (lysosomal trafficking regulator) likely do not present phenotypes of LB dysfunction, pressing unique origin and functional mechanisms of skin LBs (Boissy and Nordlund, 1997; Dell'Angelica et al., 2000).

Nonetheless, diseased models such as CEDNIK (Cerebral dysgenesis–neuropathy–ichthyosis–keratoderma) syndrome caused by a mutation in the SNARE protein SNAP29 and MEDNIK (mental retardation, enteropathy, deafness, neuropathy, ichthyosis, keratoderma) syndrome caused by S1 subunit of AP1 (AP1S1) are associated with LB dysfunction and severe ichthyosis conditions (Montpetit et al., 2008; Fuchs-Telem et al., 2011; Martinelli and Dionisi-Vici, 2014; Schiller et al., 2016). A mutation in AP1S1 is shown to be associated with defective copper metabolism caused by ATP7A and ATP7B dysfunction (Martinelli and Dionisi-Vici, 2014), suggesting a possible role of copper metabolism in LB biogenesis. ATP7A plays a critical role in melanosome biogenesis by supporting the copper-dependent activity of tyrosinase (Setty et al., 2008). Thus, I assume that cell-type-specific modulation of common molecular mechanisms contributes to LRO biogenesis.

ARC (Arthrogryposis–renal dysfunction–cholestasis) syndrome caused by a mutation in the VPS33B causes dysfunctional LBs alongside platelet alpha granule dysfunction (Gissen et al., 2004; Gissen et al., 2006; Ambrosio and Di Pietro, 2019). VPS33B interacts with VIPAR or VIPAS39 and constitutes the CHEVI (class C Homologs in Endosome-Vesicle Interaction) complex, an early endocytic machinery that helps in integrin recycling and maintaining cell polarity (Cullinane et al., 2010; Dai et al., 2016; Rogerson and Gissen, 2016; 2018). CHEVI interacts with Rab11, which further acts in a cascade of Rab3 and sec15 (Ishida-Yamamoto et al., 2007; Escrevente et al., 2021), and the intracellular trafficking is likely regulated by CLIP-170/restin along with Cdc42 and Rab7 (Raymond et al., 2008). Although these molecules are likely involved in the biogenesis of LBs, their specific functions are not yet defined.

The primary cargo glucosylceramide is transported to the maturing LBs by the ABC transporter ABCA12 localized on its limiting membrane, and accordingly, the loss of function mutations of ABCA12 blocks LB biogenesis/maturation, resulting in the fatal condition Harlequin ichthyosis (Akiyama, 2011; Scott et al., 2013). ABCG1, another ABC transporter is suggested to be involved in cholesterol transport (Jiang et al., 2010). However, the mechanisms by which other lipids and protein cargoes are packed into the maturing LBs and how they coordinate with the major cargo glucosylceramide remain unclear. Thus, cell models of this disease condition/s would help us understanding the mechanism and molecular pathways involved in LB biogenesis.

## 6 Cellular physiology is a possible dominating factor for skin LB biogenesis

Most LROs are produced in a metabolically active state of harbouring cells and coexist with housekeeping organelles. In contrast, LBs appear in an advanced differentiation state of keratinocytes just before cells entering cornification. In addition to the loss of proliferative capacity, the PM is also modified with the formation of desmosomes and corneo-desmosomes, and intracellular organelles begin to disappear (Elias et al., 1998; Proksch et al., 2008; Bikle et al., 2012; Ishida-Yamamoto et al., 2018; Mahanty and Setty, 2021). Consequently, in this condition, the maturation of LBs from already existing precursor organelles is more likely over initiation of biogenesis. The apparent absence of lysosomes and the enrichment of LBs in the SG layer further suggest that LBs may mature from lysosomes (Wolff and Schreiner, 1970; Ishida-Yamamoto et al., 2004). The functional overlap of lysosomes and LBs in epidermis barrier formation and dysfunction of both in common disease conditions further supports this idea (Holleran et al., 1994; Monteleon et al., 2018; Klapan et al., 2021; Mahanty and Setty, 2021).

We demonstrated the formation of dual-function secretory lysosomes in an *in vitro* model of human primary keratinocytes that represent the SS layer of the epidermis (Mahanty et al., 2019; Mahanty et al., 2024). These secretory lysosomes are formed through Golgi-lysosome contact, facilitated by the Golgi tethering protein GRASP65 (Golgi ReAssembly Stacking Protein of 65 kDa), which atypically localizes on the membrane of lysosomes in differentiated keratinocytes (Mahanty et al., 2024). The presence of dual-function secretory lysosomes supports our hypothetical model of LB biogenesis where we described the generation of a precursor organelle through the Golgi input, followed by their maturation into LBs upon receiving Golgi/endocytic input (Mahanty and Setty, 2021).

Under electron microscopy (EM), secretory lysosomes appear in a vacuolar or multi-vesicular morphology but lack the characteristic disc-like structures of LBs (Mahanty et al., 2024). This indicates that additional cargo/inputs are needed for LB formation, which likely occurs only at a higher differentiation stage in the SG layer. I hypothesize that either the secretory lysosomes directly mature into LBs upon receiving additional cargo with higher differentiation or the secretory lysosomes undergo exocytosis at the SS-SG junction, followed by cargo re-packaging into disks and their subsequent maturation into LBs. Cargo repackaging is logical as LBs share a large amount of lysosomal cargo and enzymes. In the latter case, the LB limiting membrane, having secretory properties, could either be derived from the Golgi apparatus or from the membrane of the secretory lysosomes, recycled after exocytosis. In both cases, input from early endosomes and additional cargoes from the Golgi will be necessary for LB maturation (Figure 2D).

## 7 Organelle disappearance linking LBs biogenesis

LBs are abundant in the SG layer cells. In contrary, conventional organelles undergo degradation in the SG layer, a process essential for the entry of SG keratinocytes into terminal differentiation/cornification (Eckhart et al., 2013; Yoshihara et al., 2015; Jaeger et al., 2019). Defects in “organelle removal” can lead to improper cornification and barrier formation, as seen in psoriasis (Eckhart et al., 2013; Akinduro et al., 2016). High levels of autophagy are reported to support organelle removal, that also support cells survival in nutrient-deficient environments, in the SG layer (Yoshihara et al., 2015; Akinduro et al., 2016). However, the absence of lysosomes in the SG layer suggests that they may transform into LBs (Wolff and Schreiner, 1970; Ishida-Yamamoto et al., 2004). This hypothesis raises several important questions: a) how does high autophagy is supported in the absence of lysosomes? b) are intracellular organelles eliminated through a different mechanism during cornification? c) is there a strict temporal regulation between these processes? d) whether artificially induced autophagy will favor LBs biogenesis *in vitro*? Nonetheless, keratinocyte-specific *Atg5* and *Atg7* knock-out mouse models do not show a cornification defect (Rossiter et al., 2013; Sukseree et al., 2013), likely suggesting that autophagy is dispensable. In any of these cases, however, the maturation of LBs in the SG layer likely temporally coordinates with organelle removal to receiving Golgi and endosomal input.

## 8 Discussion

Despite nearly six decades since their discovery (Odland, 1960), the molecular mechanisms underlying the biogenesis of LBs remain largely unknown. A significant challenge in LB research is the lack of a suitable cellular model system, which limits the use of advanced cell biology techniques, microscopy methods, and genetic tools. Our current understanding of LBs functional characteristics and cargo composition primarily comes from invaluable biochemical and electron microscopy studies conducted in the 1990s and 2000s, utilizing *in situ* skin samples and mouse models. There is debate on LB morphology as well, which further complicates our understanding of their secretion mechanisms. Developing an LB-enriched cellular model that accurately represents the SG layer is particularly difficult, given it is a physiologically modified state. Although the 3D organoid models are promising in understanding the complexities of human skin and cellular organization and may serve as alternatives to animal models, they are still limited for studying molecular interactions to study intracellular trafficking or at least at the level of sophistication it requires. Therefore, we need an appropriate cellular model and taken the unique features of LBs into account to understand their biogenesis, which is crucial for effectively targeting skin diseases. The key questions regarding the understanding of LB biology and associated drawbacks are discussed in the respective sections.
